# Tomatidine and analog FC04–100 possess bactericidal activities against *Listeria*, *Bacillus* and *Staphylococcus* spp

**DOI:** 10.1186/s40360-018-0197-2

**Published:** 2018-02-13

**Authors:** Isabelle Guay, Simon Boulanger, Charles Isabelle, Eric Brouillette, Félix Chagnon, Kamal Bouarab, Eric Marsault, François Malouin

**Affiliations:** 1 Centre d’Étude et de Valorisation de la Diversité Microbienne (CEVDM), Département de biologie, Faculté des sciences, Université de Sherbrooke, 2500 Boul. Université, Sherbrooke, QC J1K 2R1 Canada; 2 Département de pharmacologie, Faculté de médecine et des sciences de la santé, Université de Sherbrooke, 3001, 12 th avenue Nord, Sherbrooke, QC J1H 5N4 Canada

**Keywords:** Tomatidine, Aminoglycoside, Synergy, SCV, *Bacillales*, *S. aureus*, *L. monocytogenes*, Foodborne disease, Cystic fibrosis

## Abstract

**Background:**

Tomatidine (TO) is a plant steroidal alkaloid that possesses an antibacterial activity against the small colony variants (SCVs) of *Staphylococcus aureus*. We report here the spectrum of activity of TO against other species of the *Bacillales* and the improved antibacterial activity of a chemically-modified TO derivative (FC04–100) against *Listeria monocytogenes* and antibiotic multi-resistant *S. aureus* (MRSA), two notoriously difficult-to-kill microorganisms.

**Methods:**

*Bacillus* and *Listeria* SCVs were isolated using a gentamicin selection pressure. Minimal inhibitory concentrations (MICs) of TO and FC04–100 were determined by a broth microdilution technique. The bactericidal activity of TO and FC04–100 used alone or in combination with an aminoglycoside against planktonic bacteria was determined in broth or against bacteria embedded in pre-formed biofilms by using the Calgary Biofilm Device. Killing of intracellular SCVs was determined in a model with polarized pulmonary cells.

**Results:**

TO showed a bactericidal activity against SCVs of *Staphylococcus aureus*, *Bacillus cereus*, *B. subtilis* and *Listeria monocytogenes* with MICs of 0.03–0.12 μg/mL. The combination of an aminoglycoside and TO generated an antibacterial synergy against their normal phenotype. In contrast to TO, which has no relevant activity by itself against *Bacillales* of the normal phenotype (MIC > 64 μg/mL), the TO analog FC04–100 showed a MIC of 8–32 μg/mL. Furthermore, FC04–100 showed a strong bactericidal activity against *L. monocytogenes* SCVs in kill kinetics experiments, while TO did not. The addition of FC04–100 (4 μg/mL) to a cefalexin:kanamycin (3:2) combination improved the activity of the combination by 32 fold against cefalexin and kanamycin-resistant MRSA strains. In combination with gentamicin, FC04–100 also exhibited a strong bactericidal activity against biofilm-embedded *S. aureus*. Also, FC04–100 and TO showed comparable intracellular killing of *S. aureus* SCVs.

**Conclusions:**

Chemical modifications of TO allowed improvement of its antibacterial activity against prototypical *S. aureus* and of its bactericidal activity against *L. monocytogenes*. Antibacterial activities against such prominent pathogens could be useful to prevent *Listeria* contamination in the food chain or as treatment for MRSA infections.

## Background

The *Bacillales* are divided into the genus of *Staphylococcus*, *Listeria* and *Bacillus*. A number of bacterial species such as *Listeria* spp. and *Bacillus* spp. can contaminate food and cause infections in humans [1]. To name a few, *Listeria monocytogenes*, *L. ivanovii*, and *Bacillus cereus* can cause listeriosis [2] and food poisoning [3]. *Bacillus subtilis*, *B. coagulans*, *B. licheniformis* and *B. sphaericus* are also known to cause illnesses. *Bacillus anthracis* causes anthrax and can often be acquired by contact with food producing animals and cattle (beef cattle, sheep, etc.) and this bacterium is also well-known for its endospores that have been used as biological weapons [4]. Staphylococci are divided in coagulase-positive species, *Staphylococcus aureus* being the most clinically relevant of this group, and coagulase-negative species, such as *S. epidermidis*, the most prevalent pathogen associated with infections of implanted medical devices [5]. The emergence and spread of resistance to multiple antibiotics in staphylococci is now considered a real health treat and impaired therapeutic endeavor to combat these bacteria [6]. Notably, the prevalence of methicillin-resistant *S. aureus* (MRSA) has steadily increased over the recent years, not only in hospitals but also in the community [7], and in veterinary medicine and livestock [8–10].

*Staphylococcus aureus* small-colony variants (SCVs) have attracted a great deal of interest over the past recent years. *S. aureus* SCVs often present a dysfunctional oxidative metabolism causing a slow growth and a change in the expression of virulence factors [11]. This dysfunctional oxidative metabolism is also responsible for a decreased susceptibility to aminoglycoside antibiotics because this class of molecules requires the proton-motive force in order to penetrate the bacterium [12]. This respiratory deficiency is often caused by mutations affecting the electron transport system, and several SCV isolates can recover normal growth with supplemental hemin or menadione, which are needed to synthesize electron transport system components. *S. aureus* SCVs often are isolated from chronic infections, such as lung infections in cystic fibrosis (CF) patients, osteomyelitis, septic arthritis, bovine mastitis, and from infections associated with orthopedic devices [11, 13, 14]. The ability of *S. aureus* to switch back and forth from the prototypic to the SCV phenotypes in vivo is an integral part of the pathogenesis of *S. aureus* and may be responsible for the establishment of chronic infections [15, 16].

**Fig. 1:**
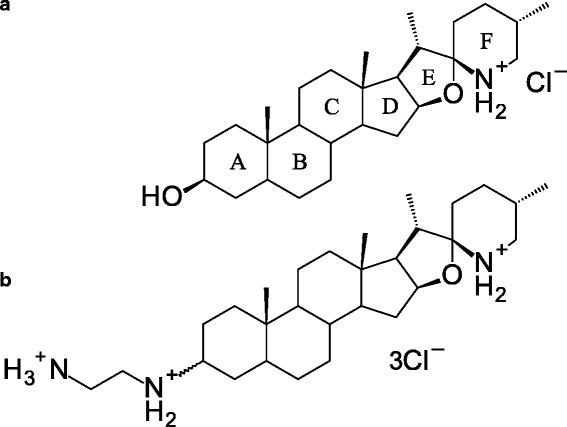
Tomatidine (**a**) is a steroid alkaloid structurally characterized by 6 rings, 12 stereogenic centers, a 3 β-hydroxyl group and spiro-fused E, F rings in the form of an aminoketal. The tomatidine analog, FC04–100 (**b**) contains a diamine in position 3 of ring A

Tomatidine (TO) is a steroidal alkaloid produced by the *Solanaceae* plant family such as the tomato [17, 18]. We showed previously the antibacterial activity of TO against *S. aureus* SCVs and also documented a strong synergic activity of TO in combination with aminoglycoside antibiotics against prototypic *S. aureus* [19, 20]. Recently, we synthesized a variety of TO analogs in order to explore the structure-activity relationship of this new class of antibiotics able to act on SCVs [21]. One analog, FC04–100, showed the same steroidal backbone as the natural molecule but with an additional carbon chain and amines on the A cycle (Fig. 1). Preliminary characterization of FC04–100 revealed an antibacterial activity that was similar to that of TO against *S. aureus* SCVs and also preserved the synergic activity with aminoglycosides against prototypic *S. aureus* [21]. However, contrary to TO, FC04–100 showed notable antibiotic activity by itself against prototypic *S. aureus* with a MIC of 8–16 μg/mL, whereas that of TO was > 64 μg/mL.

The aim of this study was to further describe the spectrum of activity of TO and FC04–100. It was of great interest to see if the discovered antibacterial activity of FC04–100 against prototypical *S. aureus* modifies or improves its spectrum of antibacterial activity. The susceptibility of several species among the *Bacillales* to TO and FC04–100 alone or in combination with other antibiotics was investigated. The improved antibacterial activity of FC04–100 was demonstrated against *Listeria monocytogenes* and methicillin-resistant *S. aureus* (MRSA).

## Methods

### Chemical reagents and antibiotics

Tomatidine (TO) (Sigma Aldrich, Oakville, Ontario, Canada) (Fig. 1a) was solubilised in dimethylsulfoxide (DMSO) at a concentration of 2 mg/mL. The TO analog, FC04–100 (Fig. 1b) was synthesized [21] and solubilised in DMSO at 20 mg/mL. Cefalexin, kanamycin, gentamicin (GEN) (all from Sigma Aldrich), were solubilised in water at a concentration of 10 mg/mL. Menadione was solubilized in DMSO, hemin in 1.4 M NH_4_OH and thymidine in water (all from Sigma Aldrich) and were prepared at a concentration of 10 mg/mL.

### Bacterial strains and growth conditions

*Staphylococcus aureus* ATCC 29213, *S. aureus* Newbould (ATCC 29740), *S. epidermidis* ATCC 12228, *Listeria monocytogenes* ATCC 13932, *Bacillus subtilis* ATCC 6333 and *Bacillus cereus* ATCC 11778 were used as prototypic strains. Methicillin-resistant *S. aureus* (MRSA) strains COL and USA100 (hospital acquired-MRSA, ATCC BAA-41) and strain USA300 (community acquired-MRSA, ATCC BAA-1556), were also used in this study. The reference laboratory strain *S. aureus* SH1000, an isogenic mutant strain derived from *S. aureus* 8523–4 but with a functional *rsb*U allele [22], was used for its good yield of biofilm production. The *S. aureus* strains CF07-L (prototypic) and CF07-S (SCV phenotype) are genetically related clinical strains originally isolated from a cystic fibrosis patient [19]. Strain CF07-S was used in the intracellular infection model. *Staphylococcus* and *Bacillus* were maintained on Tryptic Soy agar (TSA, BD, Mississauga, Ontario, Canada), whereas *L. monocytogenes* was grown on brain-heart infusion (BHI) agar (BD, Mississauga, Ontario, Canada).

### Small colony variants (SCVs)

For *S. aureus*, strain NewbouldΔ*hemB* was used as the reference SCV. NewbouldΔ*hemB* was generated from strain Newbould (ATCC 29740) by disrupting the *hemB* gene with the *ermA* cassette by homologous recombination [23]. Another SCV *hemB* mutant was similarly constructed from *S. aureus* ATCC 29213 [24]. SCVs from *B. cereus*, *B. subtillis* and *L. monocytogenes* were generated by growth in presence of a subinhibitory concentration of GEN. Briefly, overnight broth cultures (18–20 h) were used to inoculate BHI broths at a dilution of 1:100, supplemented or not with 0.25 to 1X the MIC of GEN. Cultures were incubated 18 h at 35 °C with shaking (225 rpm) and then adjusted to an *A*_595 nm_ of 2.0 in PBS at 4 °C. Determination of CFU and SCV colonies was done by serial dilution plating. SCVs were obtained by plating on TSA (BHI agar for *L. monocytogenes*) containing GEN at a concentration of 8 to 16 times the induction concentration followed by an incubation of 48 h at 35 °C. The pinpoint colonies selected by this method were confirmed to be SCVs by streaking several of them on agar plates without antibiotic. SCV that conserved their phenotype after two passages were considered to have a stable phenotype and were used in subsequent experiments. Selected SCVs had a colony size a tenth of the size of a normal colony and showed no pigmentation or hemolytic activity on blood agar plates. The SCV species were confirmed by rDNA 16S sequencing after PCR amplification using universal primers [25].

### Auxotrophy assays

For SCVs, auxotrophism is defined as the requirement of specific compounds in order to regain a normal growth phenotype [26]. An agar diffusion method was used to characterize the auxotrophism of SCVs using hemin or menadione (1–10 μg each/well) or thymidine (100 μg/well) on an inoculated Mueller-Hinton agar (MHA) or Brain-heart infusion agar (BHIA) plate [27]. Auxotrophy for specific supplements was detected by a zone of normal growth surrounding the well after 18 h of incubation at 35 °C. The results were confirmed by two independent experiments.

### Antibiotic susceptibility testing

MICs were determined by a broth microdilution technique following the recommendations of the Clinical and Laboratory Standards Institute (CLSI) [28], except that the incubation period was extended to 48 h and the medium used was BHI to allow SCVs to reach maximal growth as previously described [29]. As recommended by the CLSI, *L. monocytogenes* (and its SCVs) were grown in cation-adjusted Muller-Hinton broth (CAMHB, BD) containing 3% lysed horse blood (LHB, Remel, Hartford, CT, USA). The reported results were obtained from three independent experiments.

### Time-kill experiments

Kill kinetics were performed to determine whether the effect of compounds alone or in combination with an aminoglycoside was bacteriostatic or bactericidal. Bacteria were inoculated at 10^5^–10^6^ CFU/mL in the appropriate medium in the absence or presence of the different antibiotic compounds at concentrations specified in figure legends. At several points in time at 35 °C (225 rpm), bacteria were sampled, serially diluted, and plated on TSA for CFU determinations. Plates were incubated for 24 or 48 h at 35 °C for normal and SCV strains, respectively. The data were collected from a minimum of three independents experiments.

### Antibiotic activity in biofilms

The viability of bacteria in biofilms was evaluated based on previously described protocols [30–32]. For these assays, 96-peg lids and corresponding 96-well plates were used (Thermo Scientific, Ottawa, ON, Canada). Wells were inoculated with a suspension of *S. aureus* SH-1000 adjusted to a 0.5 McFarland standard in TSB (150 μL per wells) and plates with 96-well lids were incubated at 35 °C with an agitation of 120 rpm for 24 h. The biofilms on pegs were then washed three times with 200 μL PBS. Biofilms on pegs were further incubated in fresh 96-well plates containing 200 μL of TSB containing or not antibiotic compounds or a combination of molecules at 35 °C, 120 rpm for 24 h. The treated biofilms on pegs were washed three times in PBS and bacteria in biofilms were recovered by sonication in a new microtiter plate containing 200 μL of PBS per wells using an ultrasonicator bath for 10 min followed by centrifugation for 5 min at 1000 RPM. The bacteria recovered in each well were suspended, serially diluted, plated on TSA and incubated at 35 °C for 24 h before CFU determination.

### Cell invasion and measurement of intracellular antibiotic activity

The Calu-3 cell line (ATCC HTB 55, *Homo sapiens* lung adenocarcinoma), was cultured in Eagle’s Minimum Essential Medium (EMEM) supplemented with 0.1 mM MEM nonessential amino acids, 1 mM of sodium pyruvate, 100 U/mL penicillin, 100 μg/mL streptomycin, 2.5 μg/mL of Fungizone and 10% fetal bovine serum (FBS) at 37 °C in 5% CO_2_. For routine culture, 4 μg/mL of puromycin was added to culture media. All cell culture reagents were purchased from Wisent (St-Bruno, QC, Canada).

The cell invasion assay was performed with the Calu-3 cells in an air-liquid interface as previously described with few modifications [19]. Briefly, cells were seeded at 1.5 × 10^5^ cells per insert in a 12-well Transwell plate (Corning, Tewksbury, MA) and cultured for 10 days with the apical medium replaced each day. The complete medium in the basal compartment was replaced by the invasion medium (1% FBS and no antibiotics) 18 h before invasion assays. Inocula were prepared by suspending bacteria grown 20 h on BHI agar plates in ice-cold PBS. Bacteria were then washed three times in ice-cold PBS and suspended in the invasion medium supplemented with 0.5% BSA at a density of approximately 4.0 × 10^8^ CFU/mL. Cells were washed twice with PBS and 250 μL of bacterial suspension (multiplicity of infection [MOI] of 10, i.e., the ratio bacteria/cells) were apically added to each insert. Invasion of cells by bacteria was allowed for 3 h, inserts were emptied and washed three times with PBS. The basal medium was changed for the invasion medium with 20 μg/mL of lysostaphin (Sigma) to kill extracellular bacteria and with or without the tested antibiotic compounds. The infected cells were incubated for a total of 48 h with a change of medium at 23 h (invasion medium with lysostaphin with or without the tested antibiotic compounds). The invasion medium with lysostaphin but without tested compound was also apically added 1 h before cell lysis to ensure that only intracellular bacteria were counted. At 48 h, following three washes with PBS, the apical and basal media were removed and cells were detached with 100 μL of trypsin 0.25% and lysed for 10 min by the addition of 400 μL of water containing 0.05% of Triton X-100. Then, 50 μL of PBS (10X) was added and mixed. Lysates were serially diluted 10-fold and plated on TSA for CFU determination. Plates were incubated at 35 °C for 48 h.

### Statistical analysis

Statistical analyses were carried out with the GraphPad Prism software (v.6.02). Bacterial CFUs were transformed in base 10 logarithm values before being used for statistical analyses. One-way ANOVA and post-tests were used as appropriate for the analysis of data as specified in each of the figure legends.

## Results

### Emergence the SCV phenotype

**Fig. 2:**
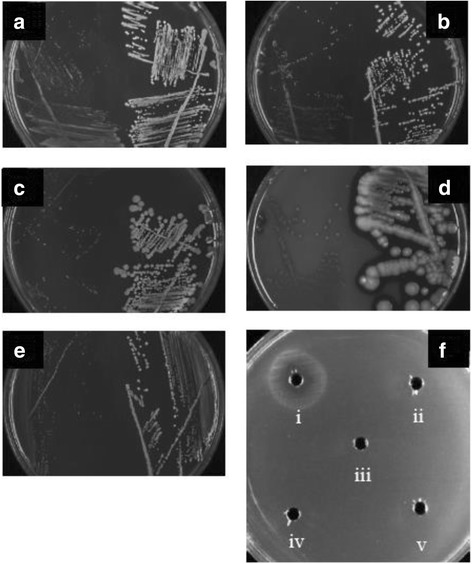
Typical colony sizes for SCV and prototypic strains (left and right sides of the plates, respectively) for (**a**) *S. aureus* CF07-S and CF07-L, (**b**) *S. aureus* NewbouldΔ*hemB* and Newbould, (**c**) *B. cereus* ATCC 11778 (SCV#1 and prototypic), (**d**) *B. subtilis* ATCC 6333 (SCV#2 and prototypic), and (**e**) *L. monocytogenes* ATCC 13932 (SCV#1 and prototypic). In (**f**), a TSA plate was inoculated with *L. monocytogenes* ATCC 13932 SCV#2 and wells were filled with (i) 10 μg hemin, (ii) 10 μg menadione, (iii) 100 μg thymidine, and diluents (iv) DMSO, and (v) NH_4_OH 1.4 N. A zone of enhanced growth is observed around (i). The enhanced growth appears white and surrounds a small zone of growth inhibition

The SCV phenotype is characterized by a slow growth yielding a small colony size on agar plates (i.e., typically 1/5 to 1/10 of the normal colony size). Two prototypic *S. aureus*, Newbould and CF07-L, and their SCV counterparts, NewbouldΔ*hemB* and CF07-S, respectively, were used as control strains (Fig. 2a and b). Figure 2 shows the colony sizes of the parental strains compared to that of the SCV derivatives. A subinhibitory concentration of GEN was used to promote the emergence of the SCV phenotype for *B. cereus*, *B. subtilis* and *L. monocytogenes*. The *B. cereus* SCVs were obtained at an inducing concentration of 1 X MIC of GEN (Fig. 2c), *B. subtilis* SCVs at 0.5 X MIC (Fig. 2d), and *L. monocytogenes* SCVs at 0.25 X MIC (Fig. 2e). The SCV isolates selected for the rest of the study were stable and kept their small-colony phenotype without a GEN selection pressure. The *B. cereus* SCVs showed auxotrophy for menadione (Table 1), some of the *L. monocytogenes* SCVs showed auxotrophy for hemin (Fig. 2f, Table 1), while the *B. subtilis* SCVs showed no apparent auxotrophy to hemin, menadione or thymidine (i.e., unknown auxotrophy, Table 1). These results show that SCVs with defects in their respiratory chain (i.e., hemin or menadione auxotrophy) can indeed emerge from a selective pressure with aminoglycosides.

**Table 1:** Susceptibility of prototypic and SCV strains and species to TO, FC04–100, GEN or the combination of GEN with one of either steroidal alkaloids

		MIC (μg/mL)				
Species and strains	Auxo^a^	TO	FC	GEN	GEN + TO (fold)^b^	GEN + FC (fold)^c^
*Staphylococcus* ssp.						
*S. aureus* ATCC 29213	–	> 64	8–16	0.5	0.06 (8)	0.06–0.12 (4–8)
*S. aureus* ATCC 29213 Δ*hemB* (SCV)	H	0.06	2	8	–	–
*S. aureus* Newbould	–	> 64	8–16	0.5	0.06 (8)	0.12 (4)
*S. aureus* Newbould Δ*hemB* (SCV)	H	0.06	2	8	–	–
*S. aureus* CF07-L	–	> 64	8	0.5	0.06(8)	0.12 (4)
*S. aureus* CF07-S (SCV)	M	0.12	2	8	–	–
*S. aureus* SH1000	–	> 64	8–16	0.5	0.06 (8)	0.06–0.12 (4–8)
*S. aureus* MRSA USA100 ATCC BAA-41	–	> 64	16	0.5	0.12 (4)	0.12 (4)
*S. aureus* MRSA USA300 ATCC BAA-1556	–	> 64	16	0.5	0.06 (8)	0.06 (8)
*S. aureus* MRSA COL	–	> 64	16	0.25	0.06 (4)	0.06 (4)
*S. epidermidis* ATCC 12228	–	> 64	8	0.5	0.12 (4)	0.12 (4)
*Bacillus* ssp.						
*B. cereus* ATCC 11778	–	> 64	8	1	0.25 (4)	0.25 (4)
*B. cereus* ATCC 11778 (SCV#1)	M	0.03	2	16	–	–
*B. cereus* ATCC 11778 (SCV#2)	M	0.03	2	16	–	–
*B. subtilis* ATCC 6333	–	> 64	8	0.12	0.03 (4)	0.06 (2)
*B. subtilis* ATCC 6333 (SCV#1)	U	0.03	2	2	–	–
*B. subtilis* ATCC 6333 (SCV#2)	U	0.03	2	4	–	–
*B. subtilis* ATCC 9372	–	> 64	8	0.12	0.015 (8)	0.06 (2)
*B. subtilis* ATCC 9372 (SCV#1)	U	0.03	1	1	–	–
*B. subtilis* ATCC 9372 (SCV#2)	U	0.03	1	1	–	–
*Listeria*						
*L. monocytogenes* ATCC 13932	–	> 64	32	0.5	0.12 (4)	0.12 (4)
*L. monocytogenes* ATCC 13932 (SCV#1)	U	0.03	1	> 64	–	–
*L. monocytogenes* ATCC 13932 (SCV#2)	H	0.03	1	> 64	–	–

*Abbreviations: TO* tomatidine, *FC* FC04–100, *GEN* gentamicin, *Auxo* auxotrophy, *SCV* small-colony variant, *H* hemin, *M* menadione, *T* thymidine, *U* unknown

^a^For SCVs, auxotrophism is defined as the requirement of specific compounds in order to regain a normal growth phenotype. An agar diffusion method was used to characterize the auxotrophism of SCVs using H, M or T. If none of these compounds restored normal growth, the auxotrophy was unknown (U)

^b^GEN was used in combination with TO at a sub-MIC of 8 μg/mL. The fold increase in GEN susceptibility is the ratio of the MIC of GEN used alone vs GEN in the presence of TO

^c^GEN was used in combination with FC at a sub-MIC of 4 μg/mL. The fold increase in GEN susceptibility is the ratio of the MIC of GEN used alone vs GEN in the presence of FC

### *Antibacterial activity of TO and analog FC04–100 against Bacillales* spp

The MICs of TO and FC04–100 against a panel of prototypic and SCV strains of the *Bacillales* are reported in Table 1. The MICs for TO ranged from 0.03 to 0.12 μg/mL against all the SCVs tested from the species *S. aureus*, *L. monocytogenes*, *B. cereus* and *B. subtilis*, whereas TO showed no activity (MIC > 64 μg/mL) against the prototypic strains including *S. epidermidis* (Table 1). Noteworthy, strains of the normal phenotype were more susceptible to GEN compared to their SCV derivatives; this was expected because aminoglycosides (e.g., GEN) require an active respiratory chain and the proton-motive force in order to penetrate the bacterium. Besides, the MICs of analog FC04–100 against the SCV strains (MIC of 1–2 μg/mL, Table 1) were slightly higher than that found for TO. On the other hand, the MICs of FC04–100 against all prototypic *Bacillales* were lower than that of TO (8–32 μg/mL for FC04–100 vs. > 64 μg/mL for TO). Furthermore, a synergy of TO or FC04–100 with GEN against all the prototypic strains tested was also observed (Table 1). Indeed, the addition of either TO or FC04–100 to GEN in the susceptibility tests provoked a 2 to 8-fold decrease of GEN MIC against all species tested (Table 1).

These results show that the spectrum of activity of TO and FC04–100 is similarly directed against the *Bacillales* (*Staphylococcus*, *Bacillus*, *Listeria*).

The interesting antibacterial activity of FC04–100 against prototypic strains was therefore noted against antibiotic multi-resistant MRSA strains (MICs of 16 μg/mL, Table 1) and such an activity was further investigated. Although the multi-resistant *S. aureus* MRSA USA100, USA300 and COL are resistant to cefalexin (beta-lactam antibiotic, MIC of 256 μg/mL) and to the aminoglycoside kanamycin (MIC of 256 μg/mL and > 1024 μg/mL, for USA100 and USA300, respectively, Table 2), the combination of both cefalexin and kanamycin decreased their respective MICs (Table 2). Indeed, the combination of cefalexin and kanamycin in a proportion of 3:2 (commercialized under the name Ubrolexin™) decreased the MIC of one or both antibiotics. This is as expected for the combination of a beta-lactam with an aminoglycoside [33]. However, the synergy is weak with MRSA strains that are resistant to one or the other or both antibiotics and the addition of 4 μg/mL of FC04–100 further improved by 8 to 32 fold the MIC of the cefalexin:kanamycin combination against these strains (Table 2).

**Table 2:** Synergistic activity of FC04–100 with the combination cefalexin:kanamycin

	MIC (μg/mL)				
Species and strains	CEF	KAN	CEF:KAN^a^	CEF:KAN (+FC)^b^	Fold^c^
*S. aureus* MRSA USA100 ATCC BAA-41	256	256	128:86	4:3	32
*S. aureus* MRSA USA300 ATCC BAA-1556	256	> 1024	256:171	8:6	32
*S. aureus* MRSA COL	256	4	4:3	0.5:0.35	8

*Abbreviations: CEF* cefalexin, *KAN* kanamycin, *FC* FC04–100

^a^CEF:KAN was used at a proportion of 3:2

^b^FC04–100 was used at a sub-MIC of 4 μg/mL

^c^The fold increase in susceptibility is the ratio of the MIC of CEF:KAN alone vs CEF:KAN in the presence of FC04–100

### Bactericidal activity of TO and analog FC04–100 against SCVs and bactericidal synergy in combination with GEN against prototypic strains

**Fig. 3:**
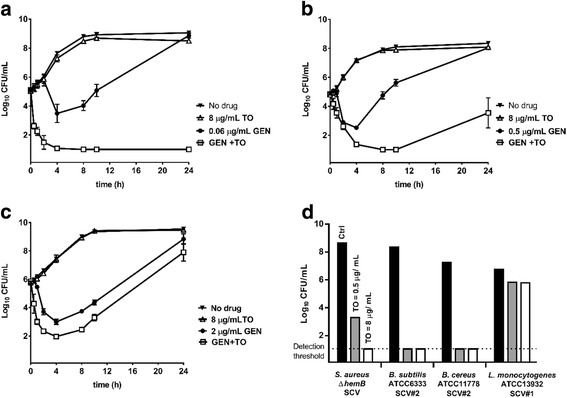
Kill kinetics of TO, GEN or the combination of both (GEN + TO) against (**a**) prototypic *B. subtilis* ATCC 6333, (**b**) *B. cereus* ATCC 11778, and (**c**) *L. monocytogenes* ATCC 13932. The bactericidal effect of TO against SCVs at 0, 0.5 and 8 μg/mL at 24 h is also shown (**d**). The results were obtained from at least three independent experiments

As was shown in Table 1, the addition of 8 μg/mL of TO to GEN reduced the MIC of GEN against all prototypic *Bacillales*. In this section, kill kinetics also show that the TO-GEN combination importantly improves the bactericidal effect of GEN against prototypic *B. subtilis* and *B. cereus* with a > 4 log10 reduction in viable bacteria at 24 h compared to the no drug control (Fig. 3a and b, respectively). However, although the MIC of GEN was improved when in combination with TO against *L. monocytogenes* (Table 1), time-kill experiments failed to demonstrate a bactericidal synergy with an improvement of at most 1 log_10_ in killing compared to that achieved with GEN alone (Fig. 3c). Similarly, the effect of TO used alone against SCVs was strongly bactericidal against *S. aureus*, *B. subtilis* and *B. cereus*, but not against the *L. monocytogenes* SCV (Fig. 3d), and this even if the MIC of TO against *L. monocytogenes* SCVs was 0.03 μg/mL (Table 1).

**Fig. 4:**
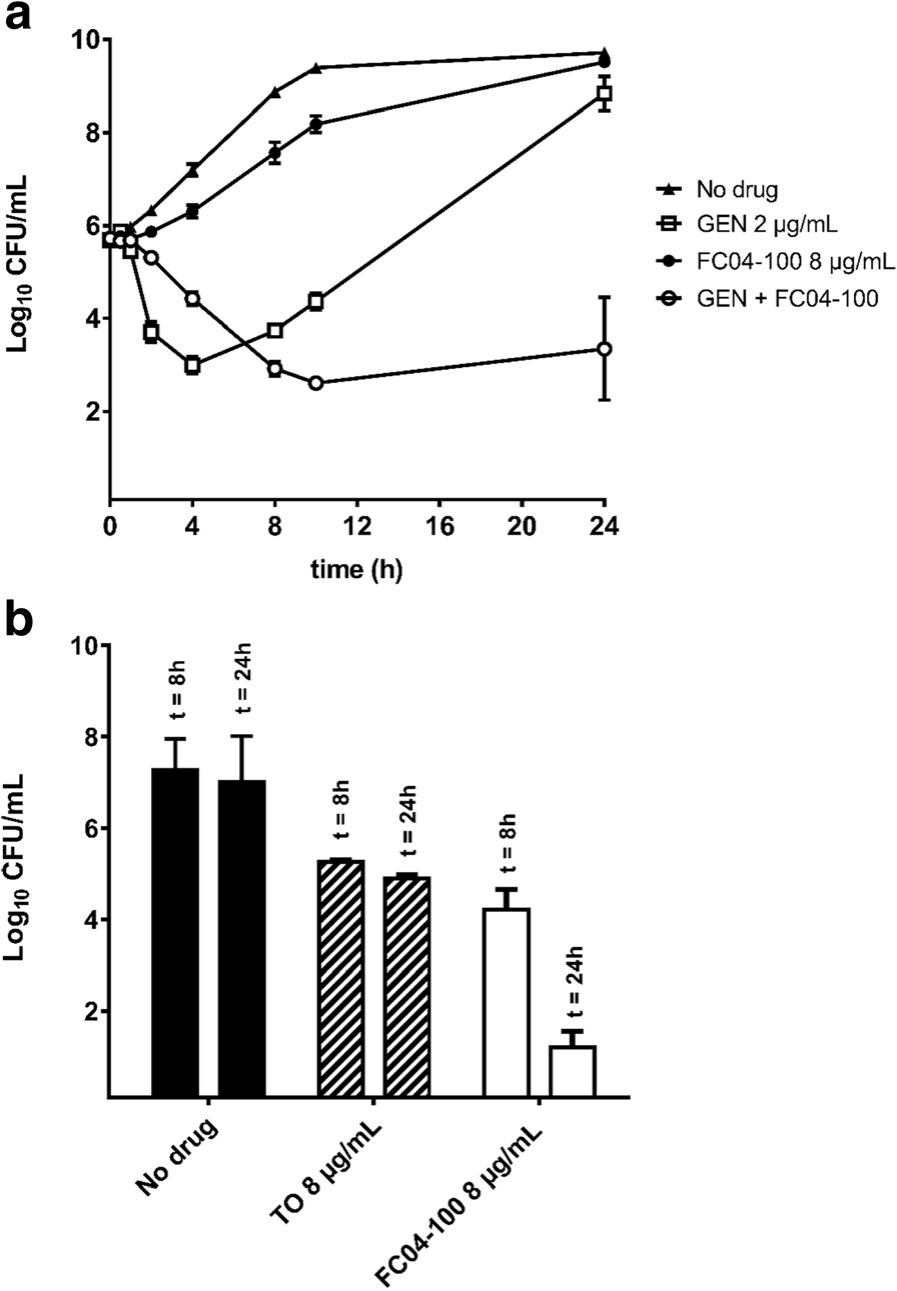
Kill kinetics of FC04–100, GEN or the combination of both (GEN + FC04–100) against (**a**) prototypic *L. monocytogenes* ATCC 13932. The bactericidal effect of TO or FC04–100 used alone against *L. monocytogenes* SCV#1 is also shown after 8 and 24 h of incubation (**b**). The results were obtained from at least three independent experiments

On the other hand, the addition of 8 μg/mL of FC04–100 to GEN (2 μg/mL) decreased by an average of 5 log_10_ the viable counts (CFU/mL) of prototypic *L. monocytogenes*, after 24 h of treatment, in comparison to GEN alone (Fig. 4a). Also, the bactericidal activity of FC04–100 used alone against the *L. monocytogenes* SCV was shown to be better than that observed for TO. To quantitate and facilitate such a comparison between TO and FC04–100, Fig. 4b shows the residual CFU of the *L. monocytogenes* SCV strain after treatment with TO or FC04–100 and calculated as the percentage of live CFU in comparison with untreated *L. monocytogenes* SCVs after 24 h of incubation. These results show that TO can be chemically modified (e.g., derivative FC04–100) to improve its antibacterial activity against *L. monocytogenes*.

### FC04–100 potentiates the activity of GEN against *S. aureus* embedded in biofilm

**Fig. 5:**
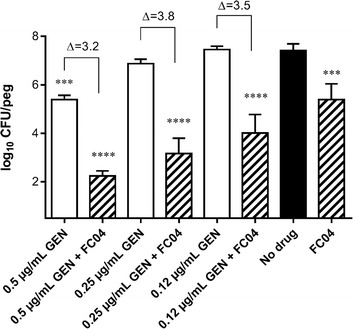
Bactericidal effect of FC04–100 alone or in combination with GEN against prototypic *S. aureus* SH1000 embedded in a biofilm. FC04–100 (FC04) was used at 4 μg/mL alone or in combination with GEN at the indicated concentrations. Data are from three independent experiments, each performed in triplicate. Significant differences in comparison to the no drug control are shown (***, *P* < 0.001 and ****, *P* < 0.0001; one-way ANOVA with a Dunnett’s post test). Data are presented as means with standard deviations

The interesting bactericidal properties of FC04–100, notably against *Listeria* (Fig. 4) prompted us to further investigate the bactericidal activity of this steroidal alkaloid in other instances where *Bacillales* spp. are difficult to kill. For example, strain *S. aureus* SH1000 produces copious amounts of biofilm and is thus suitable for use in an assay measuring the intrabiofilm bactericidal activity of antibiotics. Figure 5 demonstrates that compound FC04–100 can also kill prototypical *S. aureus* SH1000 embedded in a biofilm. Indeed, FC04–100 used alone at 4 μg/mL (i.e., 0.25–0.5 × MIC) significantly reduced the number of viable bacteria remaining in the biofilm as compared to the no drug control (*P* < 0.001). Furthermore, when FC04–100 (4 μg/mL) was used in combination with GEN, the antibacterial activity of GEN (used at either 0.25, 0.5 or 1 × MIC) was significantly improved (*P* < 0.0001). In other words, addition of FC04–100 to GEN improved its intrabiofilm bactericidal activity by more than 3 log10 at all tested GEN concentrations (Fig. 5).

### FC04–100 inhibits the intracellular replication of SCVs

**Fig. 6:**
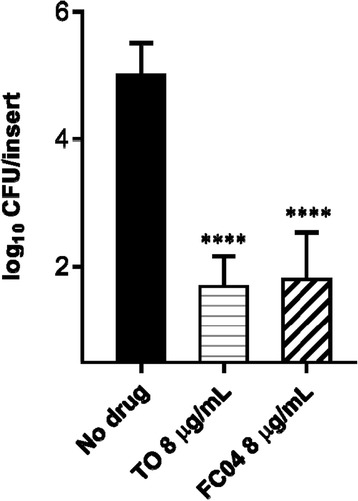
Effect of TO and FC04–100 on the intracellular load of *S. aureus* SCV strain CF07-S 48 h post-internalization. Data are from three independent experiments performed in duplicate. Significant differences among groups are shown (****, *P* < 0.0001, one-way ANOVA with Turkey’s post test). Data are presented as means with standard deviations

Another instance where some *Bacillales* spp. are difficult to kill by antibiotics is when they hide within host cells. Indeed, intracellular replication of *S. aureus* SCVs is an important contributor to the establishment of chronic infections such as those occurring in the lungs of cystic fibrosis patients. Hence, to mimic the human pulmonary epithelium, Calu-3 cells were cultivated in an air-liquid interface and were subsequently infected with the *S. aureus* SCV strain CF07-S. Strain CF07-S was originally isolated from a patient with cystic fibrosis. After invasion of cells by CF07-S, remaining extracellular bacteria were removed and Calu-3 cells were exposed to TO or FC04–100 to evaluate their ability to kill intracellular bacteria. Figure 6 shows that FC04–100 (used at 8 μg/mL), like TO, can significantly decrease the intracellular population of *S. aureus* CF07-S by at least 3 log10 compared to the no drug control (*P* < 0.0001).

## Discussion

In previous studies, we have demonstrated that TO has a strong inhibitory activity against *S. aureus* SCVs and improves the bactericidal activity of aminoglycoside antibiotics against prototypic *S. aureus*, and also more broadly, against staphylococci. The mode of action of TO and its analog FC04–100 against *S. aureus* has yet to be understood. Our initial work suggested that TO inhibits the biosynthesis of macromolecules, with a pronounced effect on protein synthesis and that when used in combination, the aminoglycoside or TO could help each other to reach their respective intracellular target through cell permeabilization [19, 20]. However, more recently, we have putatively identified the cellular target of TO as the bacterial ATP synthase using genomic analysis of TO-resistant mutants [34]. Such a cellular target would explain both the action of TO against the already impaired respiratory-deficient SCVs, the reduction in macromolecular biosyntheses resulting from a reduction in ATP production, and the synergy with an aminoglycoside which action is also linked to the respiratory chain and proton motive force. The TO-aminoglycoside synergy was documented for several prototypical strains of diverse clinical origins including aminoglycoside-resistant *S. aureus* carrying aminoglycoside modifying enzymes. This effect occurring by a mechanism that has yet to be understood was documented for several strains of diverse clinical origins including aminoglycoside-resistant *S. aureus* carrying aminoglycoside modifying enzymes. TO showed however no synergistic effect on the activity of aminoglycosides against *P. aeruginosa*, *E. coli* or *Enterococcus spp*. In the present study, we showed that the antibacterial spectrum of TO can be extended to species of the *Bacillales* order having very low MICs against *L. monocytogenes*, *B. cereus* and *B. subtillis* SCVs (0.03 to 0.12 μg/mL). Time-kill kinetics showed that the combination of TO and GEN creates a bactericidal synergy against prototypic strains of *B. subtilis* and *B. cereus*, similarly to that previously demonstrated against *S. aureus*. However, the mixture of TO and GEN did not demonstrate much improvement over the activity of GEN alone against prototypic *L. monocytogenes* in time-kill experiments. Likewise, investigations with *Bacillales* SCVs showed that TO efficiently killed *S. aureus*, *B. cereus* and *B. subtillis* but not *L. monocytogenes* SCVs despite its very low MIC and TO was thus bacteriostatic against that species. *L. monocytogenes* SCVs are indeed quite tolerant to the bactericidal effects of many classes of antibacterial agents [35]. Therefore, amelioration of the bactericidal activity of TO against this important pathogen, as seen with its derivative FC04–100, is an important breakthrough.

TO is a steroid alkaloid found in the *Solanaceae* plants [20, 21] and is structurally characterized by 6 rings, 12 stereogenic centers, a 3β-hydroxyl group on ring A and spiro-fused E, F rings in the form of an aminoketal (Fig. 1). Although we previously documented some interesting antibacterial activity for TO, the absence of an identified cellular target makes difficult the establishment of a structure-activity relationship (SAR). In initial attempts to elucidate SAR, we prepared analogs bearing modifications on ring A of TO [21]. The addition of two ammonium groups in position C3 on ring A was highly beneficial for antibiotic activity against normal non-SCV strains and the antibacterial spectrum of activity of one of such analogs, FC04–100, is detailed in the present work. On the other hand, although FC04–100 increased its antibacterial activity against prototypical strains of the *Bacillales* compared to TO (Table 1), it presents a reduced activity against *S. aureus* SCVs (from a MIC of 0.03–0.12 μg/mL for TO to a MIC of 1–2 μg/mL for FC04–100). The study of Chagnon et al. [21] also demonstrated that the stereochemistry of the 3 position substitution moderately affected activity against *S. aureus* SCVs and since FC04–100 is a mixture of stereoisomers for that position, separation and purification of FC04–100 stereoisomers will need to be done in future studies to see if one or the other stereoisomer drives the antibacterial activity against prototypical or SCV strains.

Here we demonstrated that FC04–100 was able to kill both *L. monocytogenes* SCVs as well as the normal phenotype, alone and in the presence of GEN, respectively (Fig. 4). Furthermore, FC04–100 showed a noticeable antibacterial activity on its own (MIC of 16 μg/mL) against the MRSA strains USA100, USA300 and COL (Table 1). FC04–100 was also synergistic in combination with an aminoglycoside against such MRSA strains with a 4 to 8 fold gain in the MIC of GEN. Because MRSA strains are often multi-resistant to antibiotics and often carry aminoglycoside-modifying enzymes (AMEs), we examined the possibility of using a triple antibiotic combination. Indeed, the combination of cefalexin and kanamycin in a proportion 3:2 is already approved to treat bovine mastitis pathogens under the brand name of Ubrolexin™ [36]. This antibiotic combination offers an extended spectrum of activity compared to each individual drug and is expected to cover both *S. aureus*, *Streptococcus uberis*, and *E. coli* [36–38]. Unfortunately, the increased frequency of livestock-associated MRSA [8–10] and frequent incidence of strains and species carrying AMEs [39] may limit the spectrum of activity of the combination of a beta-lactam and an aminoglycoside. With this in mind, we showed that the use of FC04–100 in combination with cefalexin and kanamycin improves by 32 fold the activity of this mixture against MRSA strains carrying AMEs (Table 2). Overall the addition of FC04–100 to the aminoglycoside-beta-lactam combination decreased the MICs of cefalexin and kanamycin below their resistance breakpoints, and this for all the MRSA strains tested.

*S. aureus*, although not primarily recognized as a typical intracellular pathogen, is able to enter and survive in host cells [40]. Moreover, the ability of *S. aureus* to convert to the SCV phenotype showing increased biofilm production, improved adherence to host cells and tissues as well as increased intracellular persistence allows this pathogen to cause chronic and difficult to treat infections [41, 42]. Discovering antibacterial drugs able to act in the intracellular compartment is generally recognized as a difficult challenge. Previously, we demonstrated that steroidal alkaloids such as TO had such an ability and could act on intracellular *S. aureus* SCVs [19], and here, we demonstrated that this was also the case for the TO analog FC04–100 (Fig. 6). Since FC04–100 showed a good bactericidal activity against *L. monocytogenes* in vitro, it will be interesting to see if it can also kill this highly specialized intracellular pathogen [43], in future studies. In addition, we also show in the present study that FC04–100 greatly improves the bactericidal activity of aminoglycosides such as GEN against bacteria embedded in biofilms. Biofilms are recognized to be a major hindrance to antibiotic action and are responsible for persistent colonization in many diseases [44] and in the food industry [45]. The important intracellular and intrabiofilm bactericidal properties of FC04–100 certainly provides further justifications for a continued interest for this class of molecules in general.

## Conclusion

We showed in this study that the spectrum of activity of TO is related to the *Firmicutes* division, and more precisely, to the order of the *Bacillales*. TO possesses an antibacterial activity against SCVs and a synergistic activity with aminoglycoside antibiotics against prototypic strains. The novel TO analog FC04–100 showed very promising new characteristics that include a much improved bactericidal activity against *L. monocytogenes* and killing of *S. aureus* when embedded in biofilms as well as when bacteria are within host cells.

## Abbreviations

AME: Aminoglycoside-modifying enzyme
Auxo: Auxotrophy
BHI: Brain-heart infusion
BHIA: Brain-heart infusion agar
CAMHB: Cation-adjusted Mueller-Hinton broth
CEF: Cefalexin
CF: Cystic fibrosis
FBS: Fetal bovine serum
FC: FC04–100
GEN: Gentamicin
KAN: Kanamycin
MHA: Mueller-Hinton agar
MHB: Mueller-Hinton broth
MIC: Minimal inhibitory concentration
MRSA: Methicillin-resistant *S. aureus*
PBS: Phosphate-buffered saline
SAR: Structure-activity relationship
SCV: Small colony variant
TO: Tomatidine
TSA: Tryptic soy agar
TSB: Tryptic soy broth
